# Identification of high-performing antibodies for Apolipoprotein E for use in Western Blot and immunoprecipitation

**DOI:** 10.12688/f1000research.133899.3

**Published:** 2023-09-29

**Authors:** Riham Ayoubi, Kathleen Southern, Carl Laflamme, NeuroSGC/YCharOS collaborative group

**Affiliations:** 1 Department of Neurology and Neurosurgery, Structural Genomics Consortium, The Montreal Neurological Institute, McGill University, Montreal, Québec, H3A 2B4, Canada

**Keywords:** Uniprot ID P02649, APOE, Apolipoprotein E, antibody characterization, antibody validation, Western Blot, immunoprecipitation

## Abstract

Apolipoprotein E is a secreted protein involved in mediating lipid distribution and metabolism among cells of specific tissues. The dysregulation of Apolipoprotein E can disturb cholesterol homeostasis, resulting in several diseases, including cardiovascular disease and Alzheimer’s disease. The therapeutic potential of Apolipoprotein E against these diseases demonstrates the importance of providing high-quality antibodies for this protein to the scientific community. In this study, we characterized fourteen Apolipoprotein E commercial antibodies for Western Blot and immunoprecipitation, using a standardized experimental protocol based on comparing read-outs in knockout cell lines and isogenic parental controls. We identified many high-performing antibodies and encourage readers to use this report as a guide to select the most appropriate antibody for their specific needs.

## Introduction

Apolipoprotein E, or APOE, is a 299 amino acid transcribed and secreted to regulate lipid homeostasis by controlling the uptake of cholesterol and lipoproteins via receptor-mediated endocytosis. ^1^^,^^2^ Three major isoforms of APOE exist, apoE4, apoE3, and apoE2. ^1^ Despite only differing by single amino acid substitutions, their functionalities are altered at both the cellular and molecular levels. ^1^ Accordingly, the binding affinity of APOE to its ligand, the LDL receptor (LDLR), varies depending on the isoform. ^2^ ApoE3 and apoE4 bind to LDLR with high affinity while apoE2 binds with low affinity. ^3^

Association studies have demonstrated apoE4 to be a genetic risk factor for cardiovascular disease, as it can cause high levels of circulating cholesterol, in the form of LDL as well as a genetic risk factor for late-onset Alzheimer’s disease. ^4^^–^^7^ The critical role of APOE in health and disease highlights the need for additional research into the protein’s mechanism of action and potential for therapeutic strategies. ^8^ Mechanistic studies would be greatly facilitated with the availability of validated and high-quality antibodies.

Here, we compared the performance of a range of commercially-available antibodies for Apolipoprotein E and validated several antibodies for Western Blot and immunoprecipitation, enabling biochemical and cellular assessment of Apolipoprotein E properties and function.

## Results and discussion

Our standard protocol involves comparing readouts from wild-type (WT) and knockout (KO) cells. ^9^^–^^11^ The first step was to identify a cell line(s) that expresses sufficient levels of Apolipoprotein E to generate a measurable signal. To this end, we examined the DepMap transcriptomics database to identify all cell lines that express the target at levels greater than 2.5 log _2_ (transcripts per million “TPM” + 1), which we have found to be a suitable cut-off (Cancer Dependency Map Portal, RRID:SCR_017655). Commercially available HAP1 cells expressed the *APOE* transcript at RNA levels above the average range of cancer cells analyzed. Parental and *APOE* knockout HAP1 cells were obtained from Horizon Discovery ( Table 1).

**Table 1.** Summary of the cell lines used.

Institution	Catalog number	RRID (Cellosaurus)	Cell line	Genotype
Horizon Discovery	C631	CVCL_Y019	HAP1	WT
Horizon Discovery	HZGHC005366c001	CVCL_SC97	HAP1	*APOE* KO (16bp deletion)

**Figure 1.**
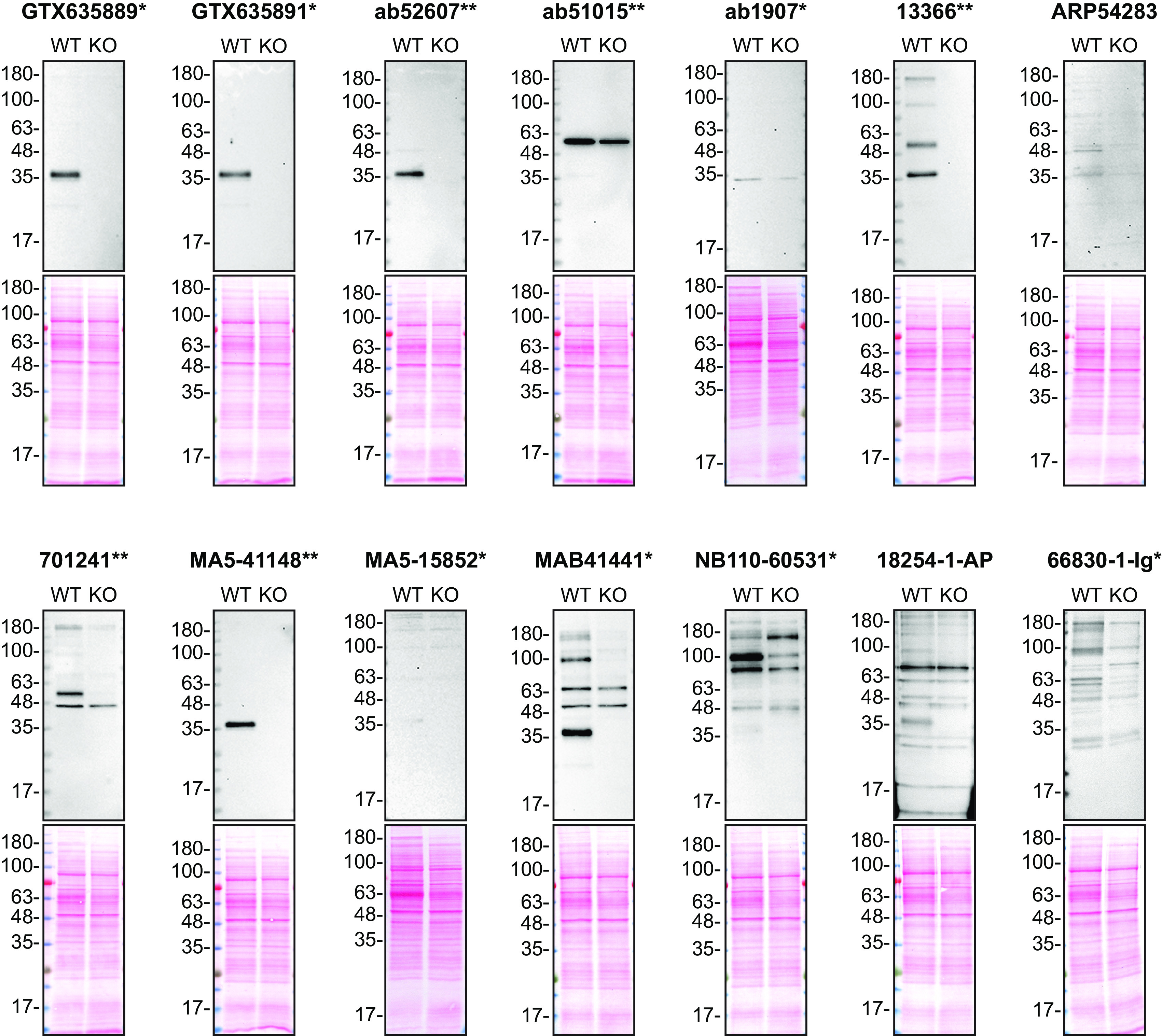
Apolipoprotein E antibody screening by Western Blot on culture media. HAP1 WT and *APOE* KO were cultured in serum free media. Media were collected, concentrated, and 30 μg of protein were processed for Western Blot with the indicated Apolipoprotein E antibodies. The Ponceau stained transfers of each blot are shown. Antibody dilutions were chosen according to the recommendations of the antibody supplier. Exceptions were given for antibodies ab1907*,13366**,18254-1-AP, MA5-15852* and 66830-1-Ig* which were titrated to the concentrations listed below, as the signals were too weak when following the supplier’s recommendations. Antibody dilutions used: GTX635889* at 1/200, GTX635891* at 1/200, ab52607** at 1/200, ab51015** at 1/1000, ab1907* at 1/200, 13366** at 1/500, ARP54283 at 1/1000, 701241** at 1/200, MA5-41148** at 1/200, MA5-15852* at 1/200, MAB41441* at 1/200, NB110-60531* at 1/200, 18254-1-AP at 1/200, 66830-1-Ig* at 1/200. Apolipoprotein E predicted band size: 36 kDa. *Monoclonal antibody, **Recombinant antibody.

**Figure 2.**
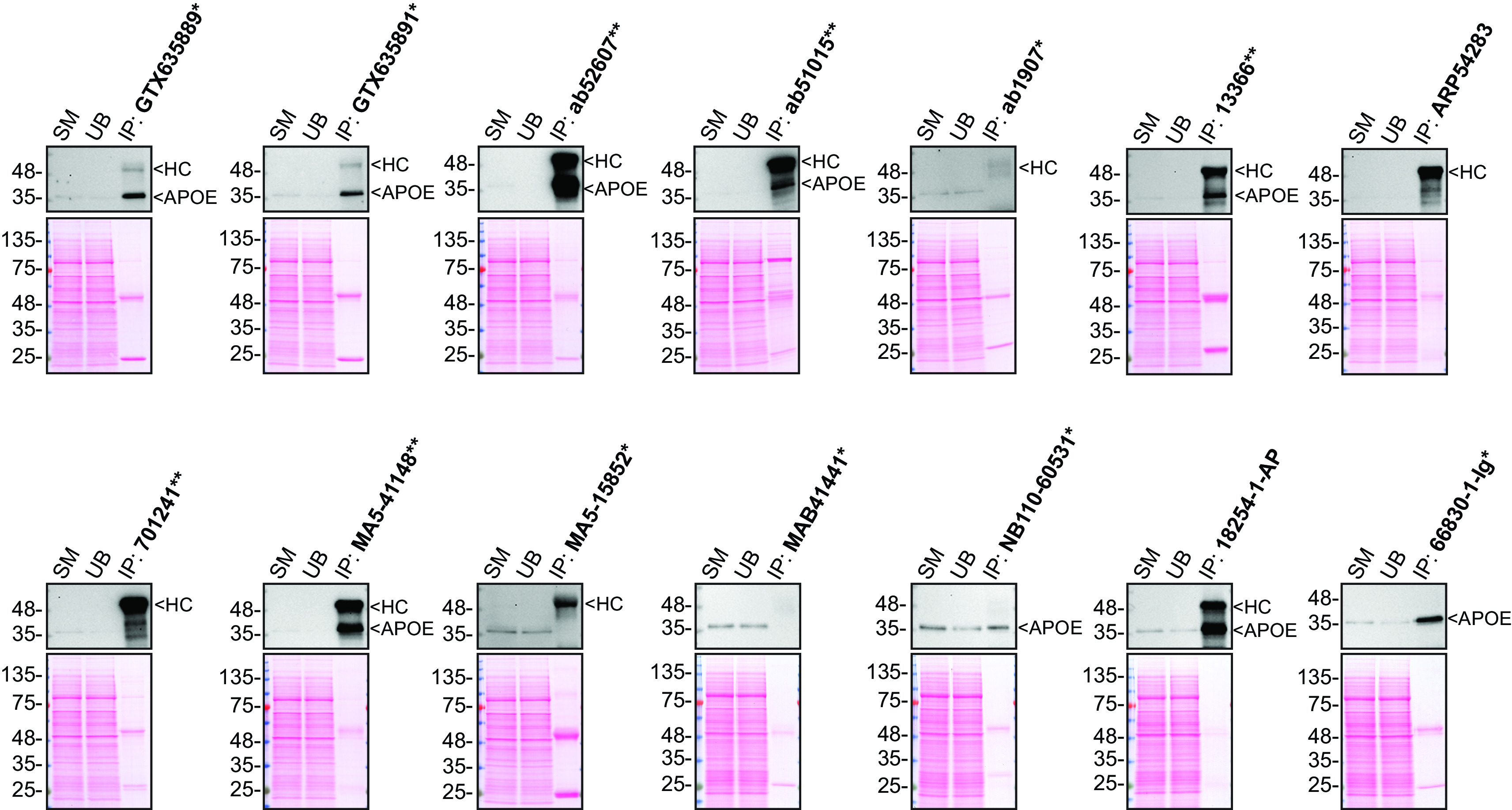
Apolipoprotein E antibody screening by immunoprecipitation on culture media. Immunoprecipitation was performed on 0.9 mg concentrated culture media from HAP1 WT, and using 2.0 μg of the indicated Apolipoprotein E antibodies pre-coupled to protein G or protein A magnetic beads. Samples were washed and processed for Western Blot with the indicated Apolipoprotein E antibody. Antibody 13366** was used at 1/500 for all Western Blots. The Ponceau stained transfers of each blot are shown. SM=3% starting material; UB=3% unbound fraction; IP=immunoprecipitate; HC=heavy chain; *Monoclonal antibody, **Recombinant antibody.

Apolipoprotein E is predicted to be a secreted protein. Accordingly, we collected concentrated culture media from both WT and *APOE* KO cells and used the conditioned media to probe the performance of the antibodies ( Table 2) side-by-side by Western Blot and immunoprecipitation. ^10^^,^^11^ The profiles of the tested antibodies are shown in Figures 1 and 2.

**Table 2.** Summary of the Apolipoprotein E antibodies tested.

Company	Catalog number	Lot number	RRID (Antibody Registry)	Clonality	Immunogenic region	Clone ID	Host	Concentration (μg/μL)	Vendors recommended applications
GeneTex	GTX635889 *	44195	AB_2909916	monoclonal	proprietary information	GT27711	mouse	1.0	Wb
GeneTex	GTX635891 *	44195	AB_2909917	monoclonal	proprietary information	GT1627	mouse	1.0	Wb
Abcam	ab52607 **	GR33789797	AB_867704	recombinant-mono	proprietary information	EP1374Y	rabbit	0.1	Wb, IP, IF
Abcam	ab51015 **	GR19880919	AB_867703	recombinant-mono	proprietary information	EP1373Y	rabbit	0.14	Wb, IP, IF
Abcam	ab1907 *	GR33619625	AB_302669	monoclonal	polymorphic amino acid 158	E6D7	mouse	1.0	IF
Cell Signaling Technology	13366 **	4	AB_2798191	recombinant-mono	proprietary information	D7I9N	rabbit	n/a	Wb, IP, IF
Aviva Systems Biology	ARP54283	QC56479-160608	AB_10640958	polyclonal	N-terminal	-	rabbit	0.5	Wb
Thermo Fisher Scientific	701241 **	2477346	AB_2532438	recombinant-mono	amino acids 240-251	16H22L18	rabbit	0.5	Wb, IF
Thermo Fisher Scientific	MA5-41148 **	XH3670137	AB_2898902	recombinant-mono	C-terminal	SC0536	rabbit	1.0	Wb
Thermo Fisher Scientific	MA5-15852 *	XH3669852	AB_11153583	monoclonal	recombinant fragment	1H4	mouse	n/a	Wb
Bio-Techne	MAB41441 *	ZRQ0318021	AB_2289763	monoclonal	recombinant fragment	395004	rat	5.0	Wb
Bio-Techne	NB110-60531 *	COEN01-2	AB_920623	monoclonal	proprietary informationn	WUE-4	mouse	1.0	Wb, IP
Proteintech	18254-1-AP	68183	AB_2878525	polyclonal	fusion protein Ag13070		rabbit	0.4	Wb
Proteintech	66830-1-Ig *	10008911	AB_2882173	monoclonal	fusion protein Ag28186	1B2C9	mouse	2.1	Wb, IF

Wb=Western Blot; IF=immunofluorescence; IP=immunoprecipitation.

* Monoclonal antibody.

** Recombinant antibody.

In conclusion, we have screened Apolipoprotein E commercial antibodies by Western Blot and immunoprecipitation and identified several high-quality antibodies under our standardized experimental conditions. The underlying data was previously uploaded to an open access repository, Zenodo. ^12^^,^^13^

## Methods

### Antibodies

All Apolipoprotein E antibodies are listed in Table 2, together with their corresponding Research Resource Identifiers (RRID), to ensure the antibodies are cited properly. ^14^ All antibodies tested detect Human ApoE. Peroxidase-conjugated goat anti-mouse and anti-rabbit antibodies are from Thermo Fisher Scientific (cat. number 65-6520 and 62-6120).

### Cell culture

HAP1 WT and *APOE* KO cell lines used are listed in Table 1, together with their corresponding RRID, to ensure the cell lines are cited properly. ^15^ Cells were cultured in DMEM high-glucose (GE Healthcare cat. number SH30081.01) containing 10% fetal bovine serum (Wisent, cat. number 080450), 2 mM L-glutamate (Wisent cat. number 609065), 100 IU penicillin and 100 μg/mL streptomycin (Wisent cat. number 450201). Cells were starved in DMEM high-glucose containing L-glutamate and penicillin/streptomycin.

### Antibody screening by Western Blot on culture media

HAP1 cells WT and *APOE* KO were washed 3× with PBS 1× and starved for ~18 hrs. Culture media were collected and centrifuged for 10 min at 500× *g* to eliminate cells and larger contaminants, then for 10 min at 4500× *g* to eliminate smaller contaminants. Culture media were concentrated by centrifuging at 4000× *g* for 30 min using Amicon Ultra-15 Centrifugal Filter Units with a membrane NMWL of 10 kDa (MilliporeSigma cat. number UFC901024).

Western Blots were performed as described in our standard operating procedure. ^16^ Midi precast 4-20% Tris-Glycine polyacrylamide gels from Thermo Fisher Scientific (cat. number WXP42012BOX) were used and proteins were transferred on nitrocellulose membranes. Proteins on the blots were visualized with Ponceau S staining (Thermo Fisher Scientific, cat. number BP103-10) which is scanned to show together with individual Western Blot. Blots were blocked with 5% milk for 1 hr, and antibodies were incubated overnight at 4°C with 5% bovine serum albumin (BSA) (Wisent, cat. number 800-095) in TBS with 0.1% Tween 20 (TBST) (Cell Signaling Technology, cat. number 9997). Following three washes with TBST, the peroxidase conjugated secondary antibody was incubated at a dilution of ~0.2 μg/mL in TBST with 5% milk for 1 hr at room temperature followed by three washes with TBST. Membranes were incubated with Pierce ECL from Thermo Fisher Scientific (cat. number 32106) or with Clarity Western ECL Substrate from Bio-Rad (cat. number 1705061) prior to detection with the iBright™ CL1500 Imaging System from Thermo Fisher Scientific (cat. number A44240).

### Antibody screening by immunoprecipitation on culture media

Immunoprecipitation was performed as described in our standard operating procedure. ^17^ Antibody-bead conjugates were prepared by adding 2 μg or 20 μL of antibody at an unknown concentration to 500 μL of Pierce IP Lysis Buffer from Thermo Fisher Scientific (cat. number 87788) in a 1.5 mL microcentrifuge tube, together with 30 μL of Dynabeads protein G - (for Mouse and rat antibodies) and protein A - (for rabbit antibodies) from Thermo Fisher Scientific (cat. number 10003D and 10002D, respectively). Pierce IP Lysis Buffer (25 mM Tris-HCl pH 7.4, 150 mM NaCl, 1 mM EDTA, 1% NP-40 and 5% glycerol) was supplemented with the Halt Protease Inhibitor Cocktail 100X from Thermo Fisher Scientific (cat. number 78446) at a final concentration of 1×. Tubes were rocked for ~1 hr at 4°C followed by two washes to remove unbound antibodies. Starved HAP1 WT culture media were concentrated as described above. 0.6 mL aliquots at 1.5 mg/L of protein were incubated with an antibody-bead conjugate for ~1 hr at 4°C. The unbound fractions were collected, and beads were subsequently washed three times with 1.0 mL of IP Lysis Buffer and processed for SDS-PAGE and Western Blot on precast midi 4-20% Tris-Glycine polyacrylamide gels. Prot-A: HRP (MilliporeSigma, cat. number P8651) was used as a secondary detection system at a concentration 0.4 μg/mL.

## Data Availability

Zenodo: Antibody Characterization Report for Apolipoprotein E, https://doi.org/10.5281/zenodo.7249055. ^12^ Zenodo: Dataset for the Apolipoprotein E antibody screening study, https://doi.org/10.5281/zenodo.7802875. ^13^
