# Supplementary material for: Identification of high-performing antibodies for Apolipoprotein E for use in Western Blot and immunoprecipitation
Source: F1000Res. 2023 Sep 29;12:810. Originally published 2023 Jul 11. [Version 3] doi: 10.12688/f1000research.133899.3 (PMC10755264; doi:10.12688/f1000research.133899.3)
Supplement: Peer review history

# Reviewer report: Reviewer response for version 3

**DOI:** 10.5256/f1000research.156691.r228353

**Published:** 2023-12-28

**Authors:** Valerio Leoni

**Affiliations:** 1 University of Milano-Bicocca, Desio, Italy

The manuscript Identification of high-performing antibodies for Apolipoprotein E for use in Western Blot and immunoprecipitation by Riham Ayoubi study the quality of commercial Anti-Apolipoporterin E antibodies.

 The Apolipoprotein E is involved in lipid distribution and metabolism among cells, especially in the CNS. The dysregulation of Apolipoprotein E can disturb cholesterol homeostasis, resulting in several diseases, including cardiovascular disease and Alzheimer’s disease. Provide high-quality antibodies for this protein to the scientific community is very important. They characterized fourteen Apolipoprotein E commercial antibodies for Western Blot and immunoprecipitation, using a standardized experimental protocol based on comparing read-outs in knockout cell lines and isogenic parental controls.

 The study was well planned and interesting.

 Results are clear,

 Figure and table informative

 A minor linguistic revision is recommended

Are sufficient details of methods and materials provided to allow replication by others?

Yes

Is the rationale for creating the dataset(s) clearly described?

Yes

Are the datasets clearly presented in a useable and accessible format?

Yes

Are the protocols appropriate and is the work technically sound?

Yes

Reviewer Expertise:

Lipidomics and metabolomics

I confirm that I have read this submission and believe that I have an appropriate level of expertise to confirm that it is of an acceptable scientific standard, however I have significant reservations, as outlined above.

# Reviewer report: Reviewer response for version 2

**DOI:** 10.5256/f1000research.153492.r201058

**Published:** 2023-09-18

**Authors:** Nathalie Macrez

**Affiliations:** 1 University Bordeaux, Bordeaux, France

The authors have compared commercially available apolipoprotein E antibodies for detection by Western blotting and immunoprecipitation using HAP1 cells expressing APOE and their KO counterparts lacking APOE. The resource is helpful but it lacks some information for full interpretation. It could be improved by addressing the following:

1. The title states "identification of high performing antibodies for APOE" - the authors indeed present some data supporting such identification but lack any interpretation of these data. Even if the authors have already answered the first reviewer about this point, the YCharOS gateway does not hinder the interpretation of results useful for the scientific community. Interpretation and limits of interpretation of the results are required and crucial for using this "report as a guide to select the most appropriate antibody for their specific needs" as stated in the abstract. The authors could give clear interpretations of their WB and IP results in terms of efficiency, specificity and potential use of these antibodies (e.g.: suitable or not for full-length APOE protein identification in WB; not suitable for immuno because recognized unspecific bands; suitable or not for identification of fragments; suitable or not for immunoprecipitation of APOE...).
2. To better understand the scope of application of the results presented in this paper, it is necessary to understand which forms of APOE are present in the cellular models studied. Which isoforms of APOE are expressed in these human cell-derived cell lines? Did the authors check the efficiency of the tested antibodies for the 3 APOE isoforms? Are these cell lines suitable for studying the cleaved forms of APOE?
3. Figure 1: As the legend states, "exceptions were given for some antibodies as the signals were too weak when following the supplier’s recommendations”. For the same reasons as done for antibodies 13366**,18254-1-AP and 66830-1-Ig*, why were the titration of ab1907* and MA5-15852* not increased? Could the low antibody titration explain the lack of APOE detection by ab1907* and MA5-15852*?
4. Could the authors explain why they chose 13366** for western blotting after IP? Did they use other antibodies for this purpose, if yes, were the results similar?

Are sufficient details of methods and materials provided to allow replication by others?

Yes

Is the rationale for creating the dataset(s) clearly described?

Yes

Are the datasets clearly presented in a useable and accessible format?

Yes

Are the protocols appropriate and is the work technically sound?

Partly

Reviewer Expertise:

neurobiology, APOE protein biochemistry, immunochemistry

I confirm that I have read this submission and believe that I have an appropriate level of expertise to confirm that it is of an acceptable scientific standard, however I have significant reservations, as outlined above.

# Response

**Published:** 2023-09-26

**Authors:** Kathleen Southern

**Affiliations:** McGill University, Canada

Dear Nathalie Macrez,

 We’d first like to thank you for taking the time to generate an in-depth review of our antibody characterization study for Apolipoprotein E. Your feedback and suggestions are appreciated as we are always looking to improve our methods and ensure our goals are clear and aligned.

 To address your first point, YCharOS is funded by various granting agencies that nominate proteins for their desired disease. We’re not experts of these proteins and prefer to remain impartial which is why we carry out the study without interpreting the results. The purpose of YCharOS is to deliver antibody characterization reports as a public good collection to benefit biomedical research as a whole. It is for this reason that we chose to publish our articles using the Data Note format, as it does not require result analysis but rather presents KO characterization data for experts of the individual targets to further their studies and projects.

 To your second point, we used DepMap, a transcriptomics database and identified wild type (WT) HAP1 as a suitable cell line as it expressed the full *APOE *transcript at a level greater than our pre-determined cut-off. An *APOE* knockout (KO) equivalent of the HAP1 cell line was purchased. Although the authors comprise of a team of experts in antibody characterization for the 3 tested applications, they are not ApoE experts and don’t know all ApoE cleaved fragments. We do know that the signals which disappear in the KO line are ApoE species. If an ApoE researcher was interested in testing the antibodies for specific isoform, we would suggest creating or purchasing a KO line for the desired ApoE isoform and see whether the signal is lost in the KO lysate compared to the WT.

 The Western blot analysis was repeated for ab1907* and MA5-15852* where both antibodies were tested at 1/200 rather than 1/1000. The results and figure legend will be adjusted and a third version will be submitted to F1000. Please note that, to begin with, Western blot is not a recommended application for ab1907,  which is why we did not increase the titration of the antibody in the original analysis.

 For immunoprecipitation, the performance of each antibody is evaluated using an antibody that specifically immunodetected ApoE in the Western blot. The 13366* antibody was able to detect both of the most prominent ApoE isoforms with high specificity, which is why it was chosen for IP.

 We hope we have answered all of your questions and concerns in this reply as well as the new version which has recently been submitted to F1000.

# Reviewer report: Reviewer response for version 2

**DOI:** 10.5256/f1000research.153492.r190833

**Published:** 2023-08-10

**Authors:** Leonard Kritharides, Maaike Kockx

**Affiliations:** 1 Department of Cardiology, Concord Repatriation General Hospital, Sydney, NSW, Australia; 2 ANZAC Research Institute, The University of Sydney, Sydney, NSW, Australia; 3 ANZAC Medical Research Institute, The University of Sydney, Sydney, NSW, Australia

We are happy with the response and approve of the article.

Are sufficient details of methods and materials provided to allow replication by others?

Yes

Is the rationale for creating the dataset(s) clearly described?

Yes

Are the datasets clearly presented in a useable and accessible format?

Yes

Are the protocols appropriate and is the work technically sound?

Yes

Reviewer Expertise:

Cell biology and biochemistry of apo E

We confirm that we have read this submission and believe that we have an appropriate level of expertise to confirm that it is of an acceptable scientific standard.

# Reviewer report: Reviewer response for version 1

**DOI:** 10.5256/f1000research.146914.r186248

**Published:** 2023-07-19

**Authors:** Leonard Kritharides, Maaike Kockx

**Affiliations:** 1 Department of Cardiology, Concord Repatriation General Hospital, Sydney, NSW, Australia; 2 ANZAC Research Institute, The University of Sydney, Sydney, NSW, Australia; 3 ANZAC Medical Research Institute, The University of Sydney, Sydney, NSW, Australia

The authors have compared and validated commercially available apolipoprotein E antibodies for detection by Western blotting and immunoprecipitation using HAP1 cells with and without apoE expression.The resource is helpful and could be improved by addressing the following:

1. Provide a summary and conclusion commenting on which antibodies are recommended for either Western Blotting or Immunoprecipitation.
2. ApoE can be cleaved generating a C-terminal and a N-terminal peptide. The current work does not investigate whether the antibodies tested detect these cleavage products. Please state the immunogenic region to which the antibodies are raised in table 2.
3. Please add details about species specificity to Table 2. If all detect human apoE please make this explicit.
4. Cellular apoE exists as non-glycosylated and glycosylated isoforms, and secreted apoE is variably but predominantly glycosylated (refs 1-3). The authors have only shown detection of secreted apoE. It would be helpful to show the ability of the antibodies to detect cellular apoE glycoforms. This will also indicate whether the antibodies display non-specific binding to other cellular proteins.
5. Provide full name for HAP1 before abbreviation is used.
6. What is the apoE genotype of the HAP1 cell line they have used?

Are sufficient details of methods and materials provided to allow replication by others?

Yes

Is the rationale for creating the dataset(s) clearly described?

Yes

Are the datasets clearly presented in a useable and accessible format?

Yes

Are the protocols appropriate and is the work technically sound?

Yes

Reviewer Expertise:

Cell biology and biochemistry of apo E

We confirm that we have read this submission and believe that we have an appropriate level of expertise to confirm that it is of an acceptable scientific standard, however we have significant reservations, as outlined above.

## References

[1] : Expression of ApoE gene in Chinese hamster cells with a reversible defect in O-glycosylation. Glycosylation is not required for apoE secretion. *J Biol Chem* .1989;264(16) :9137-40 2498327

[2] : Glycosylation of human apolipoprotein E. The carbohydrate attachment site is threonine 194. *J Biol Chem* .1989;264(15) :9094-101 2498325

[3] : Glycosylation and sialylation of macrophage-derived human apolipoprotein E analyzed by SDS-PAGE and mass spectrometry: evidence for a novel site of glycosylation on Ser290. *Mol Cell Proteomics* .2010;9(9) : 10.1074/mcp.M900430-MCP200 1968-81 10.1074/mcp.M900430-MCP200 20511397 PMC2938105

# Response

**Published:** 2023-07-21

**Authors:** Kathleen Southern

**Affiliations:** McGill University, Canada

Thank you to Leonard Kritharides and Maaike Kcokx for your extensive review of this article. We appreciate your constructive feedback and will be submitting a new version of the manuscript, to include several of your suggestions that are within the scope of this study.

 Unfortunately, we cannot comment or conclude on which antibodies to select for Western Blot and Immunoprecipitation. Previously discussed on the FAQ section of the YCharOS gateway page, we have found that, for the most part, scientists interested in our reports have the expertise to interpret the antibody characterization data. Moreover, as the antibodies are tested under one specific set of conditions, the scoring/recommendation would be valid only under the precise experimental setup and cell line used. That said, YCharOS reports serve as an invaluable guide pointing scientists to select the appropriate antibodies for their experimental needs.

 As per your second and third points, a column will be added to Table 2 in the newly submitted version of the manuscript to include the immunogenic regions of the commercial antibodies that were tested.  We also included a statement in the Antibodies sub-section of the Methods section stating that all antibodies tested were reactive against Human ApoE antigen.

 As a follow up to your fourth point, our workflow consists of testing antibodies on culture media for secreted proteins - allowing the identification of antibodies with high selectivity for the corresponding protein. We believe our data can be used to inform scientists on the best antibodies to purchase to identify intracellular ApoE. We leave it to experts in the field to optimize antibodies for their own experimental conditions. 

 For the final suggestion, we have included in Table 1 that the *ApoE* KO cell line is derived from a 16bp deletion of *ApoE*.
